# 1.5T MR-Guided Daily Adaptive Stereotactic Body Radiotherapy for Prostate Re-Irradiation: A Preliminary Report of Toxicity and Clinical Outcomes

**DOI:** 10.3389/fonc.2022.858740

**Published:** 2022-04-13

**Authors:** Francesco Cuccia, Michele Rigo, Vanessa Figlia, Niccolò Giaj-Levra, Rosario Mazzola, Luca Nicosia, Francesco Ricchetti, Giovanna Trapani, Antonio De Simone, Davide Gurrera, Stefania Naccarato, Gianluisa Sicignano, Ruggero Ruggieri, Filippo Alongi

**Affiliations:** ^1^ Advanced Radiation Oncology Department, Istituto di Ricovero e Cura a Carattere Scientifico (IRCCS) Sacro Cuore Don Calabria Hospital, Negrar di Valpolicella, Italy; ^2^ University of Brescia, Brescia, Italy

**Keywords:** MR-guided, stereotactic ablative body radiation, prostate, re-irradiation, MR-linac

## Abstract

**Background:**

Prostate re-irradiation is an attractive treatment option in the case of local relapse after previous radiotherapy, either in the definitive or in the post-operative setting. In this scenario, the introduction of MR-linacs may represent a helpful tool to improve the accuracy and precision of the treatment.

**Methods:**

This study reports the preliminary data of a cohort of 22 patients treated with 1.5T MR-Linacs for prostate or prostate bed re-irradiation. Toxicity was prospectively assessed and collected according to CTCAE v5.0. Survival endpoints were measured using Kaplan-Meier method.

**Results:**

From October 2019 to October 2021, 22 patients received 1.5T MR-guided stereotactic body radiotherapy for prostate or prostate-bed re-irradiation. In 12 cases SBRT was delivered to the prostate, in 10 to the prostate bed. The median time to re-RT was 72 months (range, 12-1460). SBRT was delivered concurrently with ADT in 4 cases. Acute toxicity was: for GU G1 in 11/22 and G2 in 4/22; for GI G1 in 7/22, G2 in 4/22. With a median follow-up of 8 months (3-21), late G1 and G2 GU events were respectively 11/22 and 4/22. Regarding GI toxicity, G1 were 6/22, while G2 3/22. No acute/late G≥3 GI/GU events occurred. All patients are alive. The median PSA-nadir was 0.49 ng/ml (0.08-5.26 ng/ml), for 1-year BRFS and DPFS rates of 85.9%. Twenty patients remained free from ADT with 1-year ADT-free survival rates of 91.3%.

**Conclusions:**

Our experience supports the use of MR-linacs for prostate or prostate bed re-irradiation as a feasible and safe treatment option with minimal toxicity and encouraging results in terms of clinical outcomes.

## Introduction

Prostate cancer is the most frequent tumor diagnosed in male population (1). The incidence of local relapses after primary external beam radiotherapy either in the definitive or post-operative setting may occur in a proportion of patients up to the 40% of cases (2).

Historically, these patients were managed with androgen deprivation therapy (ADT), as a sort of palliative treatment with a not negligible detrimental impact on quality of life (QoL) (3, 4). In recent years, there is an increasing attention towards local re-treatments in the case of previous radiotherapy (5).

Specifically in the case of cryotherapy and highly intensity-focused ultrasound (HIFU), encouraging data are reported from very small series, as these treatment approaches remain niches (6, 7).

As initially brachytherapy was the preferred option, due to the need to deliver higher doses to small volumes with the aim to spare the nearby healthy structures, more recently, stereotactic body radiotherapy (SBRT) represents an attractive non-invasive alternative in order to safely propose prostate or prostate bed re-irradiation (7, 8).

Preliminary experiences report encouraging results in terms of toxicity assessment and initial clinical outcomes (9); noteworthy, this approach is further supported by the availability of reliable imaging exams that have significantly improved the detection of the real disease burden even for lower PSA values, such as PSMA-positron emission tomography (PET) or magnetic resonance-imaging (MRI) (10).

In this scenario, the recent introduction of hybrid MRI-linear accelerators represents another helpful device for this specific setting, due to the favorable combination of a superior pelvic anatomy visualization with the possibility to daily adapt the plan based on the real-time shape and size of both target and organs-at-risk (OARs) (11).

This technology is of great interest in a setting as the re-irradiation, in which a refined identification of both target and nearby healthy structures becomes crucial in order to reduce the risk of major side effects. More specifically, available literature experiences have reported a superior outcome of SBRT both in terms of biochemical control and toxicity incidence, when compared to conventional fractionation studies (12).

In our Department we have started our clinical activity with 1.5T MR-Linac in October 2019. In the present study, approved by the local Ethical Committee on April 2019 (MRI/LINAC n°23,748), we report the preliminary results in terms of safety and efficacy for prostate and prostate bed stereotactic re-irradiation.

## Materials and Methods

This study depicts the preliminary results of the first 22 patients who received 1.5T MR-guided stereotactic re-irradiation for prostate cancer after previous definitive or post-operative radiotherapy. In all cases, patients had only local relapse with no evidence of regional or distant relapses.

All patients were treated with 1.5T MR-Linac Unity (Elekta, Stockholm, Sweden).

Inclusion criteria for the purpose of this study were: radiological evidence of local recurrence after PSA rising (PSA value: nadir + 2 ng/ml for definitive RT, or an increase above 0.2 ng/ml for post-operative RT) detected by means of MRI, Ga-PSMA or Choline-PET performed depending on PSA levels, a minimum interval of 12 months from the previous radiotherapy course, International Prostate Symptom Score (IPSS) <10, Karnosky Performance Status (KPS) ≤70, and specific written informed consent. A re-biopsy was not considered as mandatory. Patients’ characteristics are summarized in **Table 1**.

**Table 1:** Patient characteristics.

Characteristic	N
**Median age**	66 years (51-85)
**Risk Group**	
*Low risk*	3
*Intermediate risk*	7
*High risk*	12
**Median time interval between RT courses**	72 months (12-1460)
**Median PSA pre-reSBRT**	1.7 ng/ml (0.34 - 8.58 ng/ml)
**Site of recurrence**	
*Prostate bed*	10/22
*Prostate*	12/22
**Re-SBRT dose**	30 Gy/5 fractions
**Concurrent ADT**	4/22

### Radiotherapy Procedures

For the simulation process, patients were educated to have a comfortably full bladder (to drink 500cc of water 15-20 minutes before the scan) and to have an empty rectum (to use a fleet enema 2 hours before the scan). The same protocol was applied prior to each fraction. For all patients, a 3mm slice thickness pelvis-CT was acquired in supine position for dose calculation purposes. Afterwards, a T2-weighted gradient-echo was acquired in the same position using the KneeSTEP and FeetSTEP MR-compatible devices (Elekta, Stockholm, Sweden). As a part of the positioning process, the coil is positioned anteriorly and fixed to the table (13).

Regarding clinical target volume (CTV) delineation, the clinical target volume consisted of the entire prostate gland or of the PET-positive area within the prostate bed in the post-operative setting.

The planning target volume (PTV) was generated by applying to the CTV a 3-5 mm margin in all directions. The following structures were delineated as organs at risk (OARs): rectum, bladder and prostatic urethra, penile bulb and femurs.

Our planning objectives were to have a dose distribution normalized to guarantee a minimum 95% of the PTV coverage by at least the 95% of the prescribed dose, with less than 2% of the PTV to receive the 107% of the prescribed dose. Intensity modulated radiotherapy (IMRT) offline plan optimization was performed applying 16 static fields in step-and-shoot modality. The same approach was used for daily online ‘adapt-to-shape’ (ATS) workflow. For the OARs, the following constraints were applied for baseline treatment planning and for all the daily-adapted sessions: V10<40%, V18<20% for rectum; V10<25%, V18<15% for bladder; Dmax<30Gy for urethra; V24<10% for femurs; V24<50% for penile bulb (14).

The daily-adaptive workflow for Elekta Unity is based on two alternative strategies: the ‘adapt-to-position’ (ATP) and ATS methods. The ATP workflow mainly consists of a daily update of the isocenter position, and it does not require daily re-contouring. For ATS, a full re-contouring of both target and OARs is performed on the daily MRI, and afterwards a full re-planning is performed based on the anatomy of the day.

A detailed description of the daily procedure for prostate SBRT has already been reported in a previous study (15).

Briefly, prior to each fraction a T2-weighted MRI (pre-MRI) is acquired and rigidly fused with the baseline planning MRI. The original set of structures is projected onto the daily pre-MRI and edited as necessary by the physician. Then, the plan is fully re-calculated and optimized. Afterwards, a second verification MRI is acquired to check on any deformation of bladder and rectum. In the case of not negligible deformations, the patient is invited to repeat the entire procedure, otherwise the treatment is delivered using a cine MRI in coronal and sagittal planes to assess organ motion during the delivery phase.

### Toxicity and Quality of Life Assessment

Acute and late toxicity data were prospectively collected and assessed using the Common Terminology Criteria for Adverse Events (CTCAE v5,0), assuming as acute any adverse event occurring within 90 days from the end of treatment, and as late any adverse event occurring after 90 days from the end of treatment. For all patients, after the end of SBRT, the first follow up was scheduled after 60 days, and then every three months for the first year.

### Statistical Analysis

Descriptive statistics were collected for baseline patients’ characteristics.

Toxicity assessment was the primary endpoint of the study, while secondary endpoints were: biochemical relapse-free survival (BRFS), distant progression-free survival (DPFS) and overall survival (OS). Survival estimates were performed with the Kaplan-Meier method Statistical analysis was performed using Medcalc v20.023 (MedCalc Software Ltd – Ostend, Belgium).

## Results

### Patients’ Characteristics

From October 2019 to October 2021 a total of 22 patients received 1.5T MR-guided stereotactic body radiotherapy for prostate or prostate-bed re-irradiation. In 12 cases SBRT was delivered to the prostate after primary curative EBRT in 10 patients (including one case who received curative SBRT as first treatment) and brachytherapy in 2 patients. The remaining 10 patients received MR-guided SBRT to the prostate bed after previous post-operative conventional radiotherapy (respectively 4 adjuvant and 6 salvage RT). The median interval between the two courses of RT was 72 months (range, 12-1460), with local relapse detected by means of Choline-PET in 5 patients, PSMA-PET in 15 patients and MRI scan in 4 patients. Median pre-SBRT PSA value was 1.7 ng/ml (range, 0.34-8.58 ng/ml). SBRT treatment was delivered concurrently with ADT in 4 cases, with all patients who were already ongoing with systemic treatment. Median CTV and PTV were respectively 11.65 cc (range, 0.8-30.3 cc) and 23.3 cc (range, 4.8-64.2 cc), with no statistically significant variations of PTV volume between the sessions. All patients received a total dose of 30 Gy in 5 sessions delivered on alternate days in 19 patients and on consecutive days in 3 patients.

### Toxicity

All patients completed the scheduled treatment with no interruptions. Acute toxicity rates were as follows: for genitourinary (GU) adverse events, we recorded G1 in 50% (n=11), and G2 in 18% (n=4); urinary tract pain and urinary obstruction were the most frequent side effects; for gastrointestinal (GI) adverse events, G1 toxicity was observed in 31.8% (n=7) of cases, while G2 events occurred in 18% (n=4) of patients.

With a median follow-up of 8 months (range, 3-21), for late toxicity, we have recorded G1 and G2 GU events respectively in 50% (n=11) and 18% (n=4) of cases. For GI toxicity, G1 events were reported in 27% (n=6) of cases, while G2 in 13.6% (n=3) of patients. No acute or late G3 or higher GI/GU events occurred. (**Tables 2**–**4**).

**Table 2:** Acute (A) and late (B) toxicity patterns for the entire population.

A	Genitourinary	G1	G2	
	Urinary Tract Pain Urinary	7	2	
	Urgency	3		
	Urethral Stenosis	1	2	
**A**	**Gastrointestinal**	**G1**	**G2**	
	Diarrhea			
	Rectal Tenesmus	5	4	
	Rectal Proctitis	2		
**B**	**Genitourinary**	**G1**	**G2**	**G3**
	Urinary Tract Pain Urinary	7	2	
	Urgency		2	
	Urethral Stenosis	4		
**B**	**Gastrointestinal**	**G1**	**G2**	**G3**
	Diarrhea	4		
	Rectal Tenesmus	2	3	
	Rectal Bleeding			

**Table 3:** Acute toxicity patterns for prostate and prostate bed re-irradiation.

Prostate	Genitourinary	G1	G2	G3
	Urinary Tract Pain Urinary	4	1	
	Urgency	3		
	Urethral Stenosis	1	2	
**Prostate**	**Gastrointestinal**	**G1**	**G2**	**G3**
	Diarrhea			
	Rectal Tenesmus	3	3	
	Rectal Proctitis	1		
**Prostate bed**	**Genitourinary**	**G1**	**G2**	**G3**
	Urinary Tract Pain	3	1	
	Urinary Urgency			
	Urethral Stenosis			
**Prostate bed**	**Gastrointestinal**	**G1**	**G2**	**G3**
	Diarrhea			
	Rectal Tenesmus	2	1	
	Rectal Proctitis	1		

**Table 4:** Late toxicity patterns for prostate and prostate bed re-irradiation.

Prostate	Genitourinary	G1	G2	G3
	Urinary Tract Pain	4	2	
	Urethral Stenosis		2	
	Urinary Urgency	1		
**Prostate**	**Gastrointestinal**	**G1**	**G2**	**G3**
	Diarrhea	3		
	Rectal Tenesmus	1	3	
	Rectal Bleeding			
**Prostate bed**	**Genitourinary**	**G1**	**G2?**	**G3**
	Urinary Tract Pain	3	2	
	Urethral Stenosis			
	Urinary Urgency	3		
**Prostate**	**Gastrointestinal**	**G1**	**G2**	**G3**
	Diarrhea	1		
	Rectal Tenesmus	1		
	Rectal Bleeding			

### Clinical Outcomes

**Figure 1:**
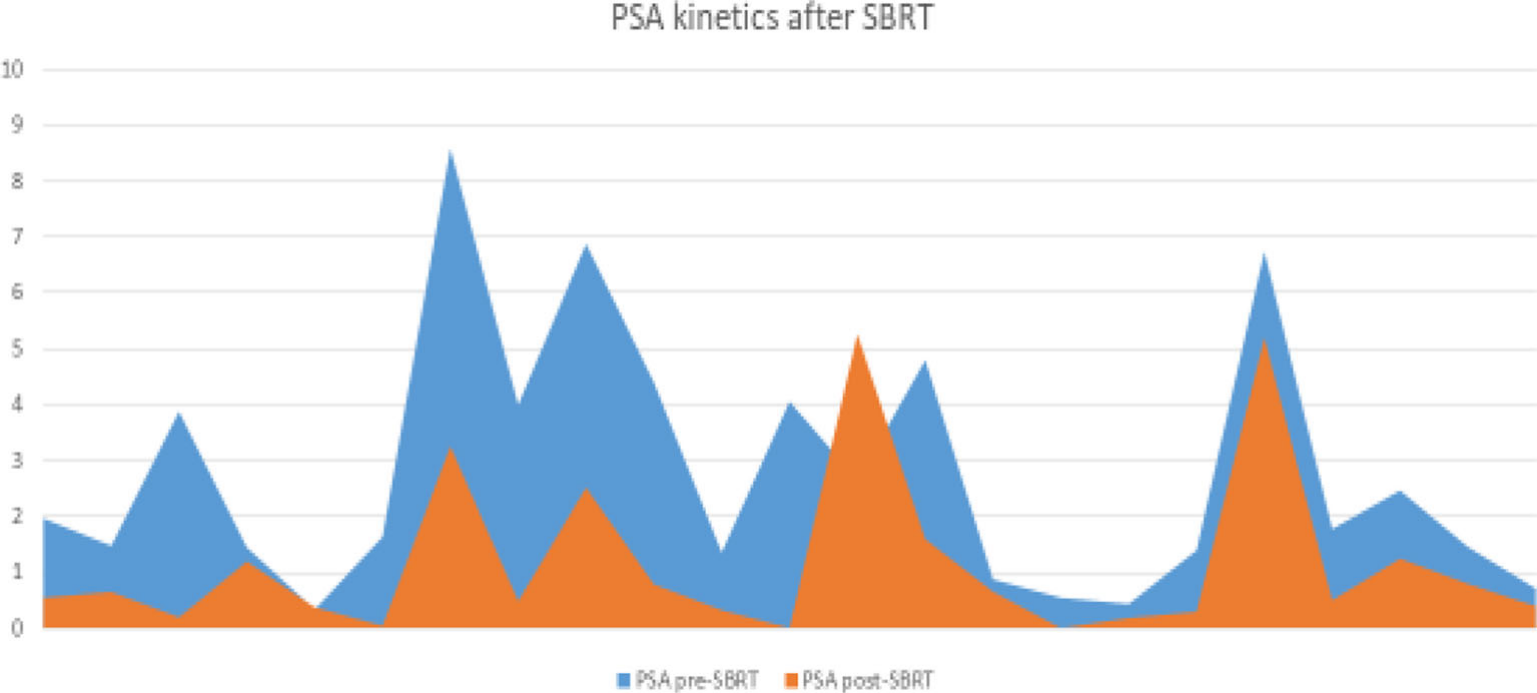
PSA kinetics after SBRT.

**Figure 2:**
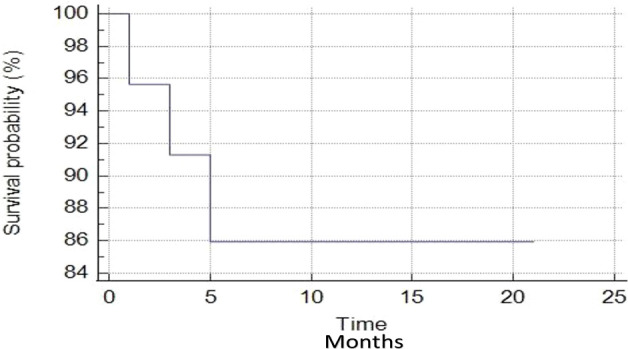
Biochemical relapse-free survival curve.

**Figure 3:**
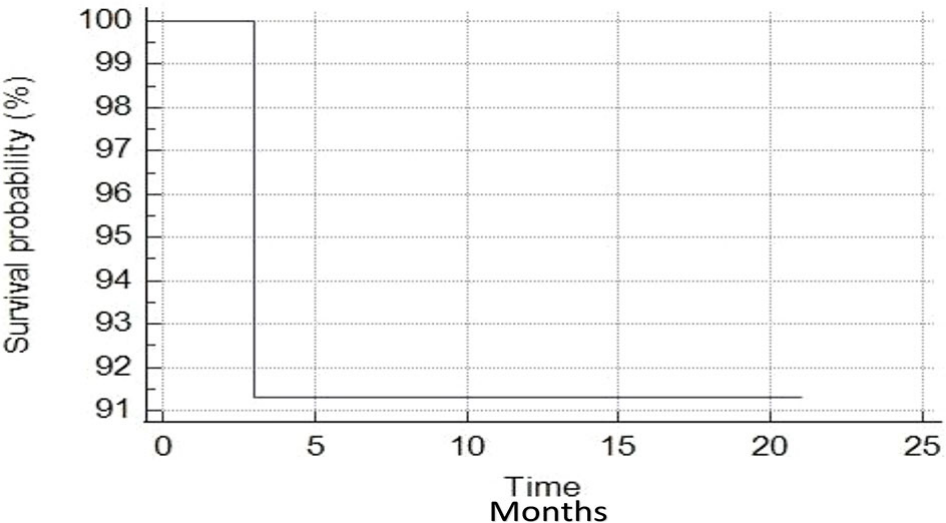
Androgen deprivation therapy-free survival curve.

All patients are currently alive, with no death occurred until the last follow-up. The median PSA-nadir value after MR-guided SBRT was 0.49 ng/ml (range, 0.08-5.26 ng/ml) (**Figure 1**). For all patients, biochemical failure was associated with a radiological disease progression, with 1-year BRFS and DPFS rates of 85.9%. Three patients developed a biochemical and radiological failure, with two of them candidate to ADT due to the evidence of polymetastatic spread. The remaining one received a further SBRT treatment to the lymph-nodal site of oligoprogression. Twenty patients remained free from ADT until the last follow-up with 1-year ADT free survival rates of 91.3%. (**Figures 2**, **3**).

## Discussion

In the present experience we have reported the preliminary outcomes of a cohort of 22 patients who received stereotactic re-irradiation for prostate or prostate bed local relapses treated by means of 1.5T MR-guided daily-adapted RT. Due to the relative novelty of this technology, there is a lack of literature data reporting the outcomes of patients treated with hybrid MR-linacs.

Recently, Michalet et al. (16) published preliminary data regarding the first 20 patients with isolated prostate or prostate bed recurrence after previous radiotherapy, who received stereotactic re-irradiation by means of 0.35T MR-Linac. In this study, preliminary toxicity assessment was promising with no evidence of G3 toxicity, although follow-up was quite limited and several fractionation regimens were applied.

Compared to the abovementioned experience, in our series there is a substantial homogeneity in dose prescription with all the patients receiving 30 Gy in 5 fractions, as it represents the most frequently adopted schedule reported in the literature (17). In agreement with the other MR-guided SBRT study, no G3 acute or late event was recorded, supporting the promising toxicity profile of this treatment, and highlighting the potentially favorable impact of this technology in refining the accuracy and precision for SBRT re-irradiation.

The safety profile of prostate re-irradiation was also found in a previous experience of our Department concerning 24 patients treated with conventional linacs (18); however, the use of daily-adapted radiotherapy with real-time replanning may result in superior organs-at-risk sparing and improved target coverage. This was also reported in a previous study of comparison between MR-guided SBRT and conventional linac-based SBRT for curative prostate cancer treatment, resulting in a lower rate of constraint violations in the cohort of patients treated with 1.5T MR-Linac (19).

The favorable toxicity pattern of the present study is in agreement with the available literature evidence: when compared to other treatment modalities such as radical prostatectomy, as reported in the MASTER meta-analysis, HDR- and LDR-brachytherapy along with SBRT have been described as the techniques collecting a lower incidence of severe GI and GU adverse events (5).

In our series, no re-biopsy was performed for a histological confirmation of recurrence. As also stated in the ESTRO-ACROP consensus, this issue remains a matter of debate as some Authors support the reliability of modern metabolic and morphologic imaging as a trustworthy surrogate of pathological confirmation (6).

Also the optimal total dose remains a matter of debate, with some Authors hypothesizing a potential radiosensitizer effect of ADT; therefore, we decided to apply the most commonly adopted fractionation regimen according to other literature experiences and institutional previous studies (20). Nonetheless, given the constantly growing attention towards this treatment option and the encouraging results recorded to date, future phase I-II trials may provide stronger evidence to identify the optimal dose to achieve a longer ADT-free interval.

Concerning the target volume delineation in the case of prostate re-irradiation, we decided to treat the entire gland in light of the multifocal nature of prostate cancer, although some experiences favorably report the role of focal re-irradiation as a means to achieve improved toxicity outcomes (21).

As far as clinical outcomes, keeping in mind the limited follow-up, our data are in agreement with previously published experiences, supporting the role of re-irradiation as an effective alternative to the premature start of ADT, with only 2 patients that received ADT after the SBRT treatment.

The present study has some limitations: first, the small sample size of the cohort affects the power of the evidence, secondly, the follow-up is relatively short. Nonetheless, this experience represents the largest series of patients treated with MR-guided daily adapted stereotactic re-irradiation for prostate cancer.

## Conclusions

Our experience supports the use of MR-linacs for prostate or prostate bed re-irradiation as a feasible and safe treatment option with minimal toxicity and encouraging results in terms of clinical outcomes. More mature data are warranted in order to further confirm the preliminary data of this study.

## Data Availability Statement

The raw data supporting the conclusions of this article will be made available by the authors, without undue reservation.

## Author Contributions

Conception of the study: FC, MR, FA. Drafting of the manuscript: FC, MR, VF, RM. Data collection: LN, FR, NGL, GT. Data analysis: DG, SN, GS, ADS, Manuscript editing: RR, FA.

## Conflict of Interest

The authors declare that the research was conducted in the absence of any commercial or financial relationships that could be construed as a potential conflict of interest.

## Publisher’s Note

All claims expressed in this article are solely those of the authors and do not necessarily represent those of their affiliated organizations, or those of the publisher, the editors and the reviewers. Any product that may be evaluated in this article, or claim that may be made by its manufacturer, is not guaranteed or endorsed by the publisher.
